# How did providers of home care for older adults manage the early phase of the Covid-19 pandemic? A qualitative case study of managers’ experiences in Region Stockholm

**DOI:** 10.1186/s12913-023-10173-8

**Published:** 2023-10-27

**Authors:** Janne Agerholm, Bo Burström, Pär Schön, Ann Liljas

**Affiliations:** 1 Aging Research Center, Department of Neurobiology, Care Sciences and Society, Karolinska Institutet, Stockholm, Sweden; 2 Department of Global Public Health, Karolinska Institutet, Stockholm, Sweden; 3 Department of Social Work, Stockholm University, Stockholm, Sweden

**Keywords:** Covid-19, Home care services, Older adults, Health care professionals, Pandemic, Care provider

## Abstract

**Background:**

In the spring of 2020, the Covid-19 outbreak sent a shock wave through the Swedish society and placed an extraordinary pressure on the health and social care system for older people. In the initial phase there were few guidelines for care providers to follow and staff in home care organisations often had to tackle challenges posed by the pandemic as they appeared. The aim of this study was to understand how the spread of Covid-19 was managed in organisations providing home care to older adults in different municipalities in Region Stockholm, and what actions were taken to minimise the spread of the disease among clients and staff.

**Method:**

A descriptive qualitative study was performed based on eight interviews with managers of home care providers for older adults in three different municipalities in Region Stockholm.Three of the eight providers operate within an integrated care system. Data were analysed using conventional content analysis.

**Results:**

Three themes were identified covering actions taken to handle the spread of the virus, feelings of insecurity and anxiety, and internal and external factors influencing how the pandemic was tackled. There was no single strategy followed by all municipalities or organisations, however, there were similarities between the organisations. One such example was the introduction of cohort care and the experience of lacking personal protective equipment. Providers in the integrated care system emphasized some advantages with their system that was seen as facilitators for minimising the risk of spreading the virus, like the joint meetings with managers from both health and social care and the close contact with healthcare professionals in relation to dissemination of hygiene instructions.

**Conclusion:**

Social care workers providing home care to older persons are an important group in preventing dissemination of infectious diseases like Covid-19. For better readiness and preparedness for future pandemics, municipal home care services would need larger stocks of personal protective equipment, clear guidelines and more training on how to reduce dissemination of disease. Ways to achieve closer communication between health and social care providers should also be investigated.

**Supplementary Information:**

The online version contains supplementary material available at 10.1186/s12913-023-10173-8.

## Introduction

 In the spring of 2020, the Covid-19 outbreak sent a shock wave through the Swedish society and placed an extraordinary pressure on the health and social care system for older people, as in most other countries around the globe. At the initial phase of the pandemic, Sweden had similar objectives and strategies as other countries: slow the spread of the virus (i.e., “flatten the curve”) and protect vulnerable groups, particularly older persons. However, soon it emerged that the Swedish Covid-19 strategy stood out from other countries. Unlike many other nations, Sweden did not respond with a general lockdown and other harsh restrictions. Instead, the Swedish strategy was based on voluntary measures, with an emphasis on individual responsibility to follow governmental recommendations [1–3]. Further, there were no official routines and guidelines for handling the Covid-19, except for regular hygiene routines. In June 2020, the Swedish government appointed an independent inquiry committee – the Corona Commission – to review the Swedish Covid-19 strategy [2, 4, 5] which found that the Swedish healthcare system was not prepared and not equipped to cope with the increased pressure caused by the pandemic and it took a long time to adapt to the situation and develop routines.

The responsibility for healthcare and social care services in Sweden is highly decentralised and divided between three tiers of government: the national level, mainly responsible for legislation; the regions, which are responsible for organising and providing healthcare, and the municipalities, responsible for organising and providing social care and to some extent home based healthcare. However, in Region Stockholm home healthcare is a responsibility of the region. The decentralisation of home care services gives the municipalities a high degree of self-governance when it comes to how services are organised and delivered in each municipality. In Norrtälje municipality in Region Stockholm, health and social care for older people has been integrated into the same organisation, whereas in the other municipalities in Region Stockholm health and social care is divided between the region and the municipalities. This division between responsibilities for health- and social care, imposed challenges during the Covid-19 outbreak as municipalities were not prepared for a pandemic. The actions taken to tackle the Covid-19 outbreak came to some extent too late or were insufficient to protect older citizens against the virus. A report has also shown that in the initial phase of the pandemic managers and staff in care homes and home care organisations felt they were left to themselves to tackle the crisis [2, 6].

Research has shown that managers of care organisations have an essential role ensuring high care quality and patient safety [7]. The pandemic forced managers to handle new and often unforeseen tasks and situations. Even though the pandemic particularly affected older adults and managers faced numerous challenges related to care provided to this population segment, little research has focused on managers in home care. One of few studies is an interview study of 13 Norwegian managers of care homes and home care services focusing on leadership and management strategies during the Covid-19 pandemic. The managers reported having faced challenges such as insufficient plans for infection control, constant changes to guidelines, lack of staffing, and challenges related to poor communication and information flow. However, the views of the managers of home care organisations were not presented separated from the other participants’ views, and the primary focus of the study was to report on resilience strategies [8]. An American study of 37 interviews with home care staff and clients of which 12 interviewees were managers, reported similar challenges as the Norwegian study. Yet, the American managers also reported on challenges with retaining clients as some suspended home care, challenges on how they could prioritise personal protective equipment for staff, and challenges with the management of symptomatic and infected clients and staff [9]. The managers’ concern protecting their staff has also been reported in a survey completed by 94 managers of home care organisations across the USA [10]. The study reported that staffing was a constant challenge due to staff being symptomatic, Covid-19 positive and/or in quarantine.

Studies focusing on the management of Covid-19 in the home care setting has been highlighted as a research field of particular importance to refine strategies for infection prevention and control measures in the home [11]. This is the first Swedish study that examines the views of managers of home care services. In addition, it is also the first study to explore such views from managers of home care organisations operating in an integrated care setting.

Norrtälje municipality is a unique example of an integrated health and social care organisation. The Norrtälje model combines the regional and municipal governance structures into a joint comprehensive health and social care organisation with a high degree of financial and structural integration. The organisation is steered by a board consisting of politicians from the Region Stockholm and Norrtälje municipality (for a thorough description of the Norrtälje model see for example [12, 13].

The aim of the integrated care model in Norrtälje is to achieve both vertical and horizontal integration in the organisation and the model is characterised by having a focus on health promotion for the population, as well as integrating the health and social care organisation on the administrative level [12]. The features of the Norrtälje model may enable to overcome several of the structural problems and barriers highlighted by the Corona Commission and facilitate a more sufficient coping with the challenges posed by the Covid-19 pandemic.

The aim of this study is to understand how the spread of Covid-19 was managed in organisations providing home care to older adults in different municipalities in Region Stockholm during the first wave of the pandemic. What actions were taken to manage the new situation and minimise the spread of the disease among clients and staff and what could have been done differently?

## Methods

This is a descriptive qualitative study [14, 15] based on seven interviews with managers of home care providers for older adults in Region Stockholm, Sweden (ethical approval 2020-04577). The descriptive design was choosen as descriptive qualitative studies is “especially amenable to obtain straight and largely unadorned answers to questions of special relevance to practitioners and policy maker” [14] and to “uncover the ‘who, what and where’ of events or experiences” [16]. In this study, we want to uncover what actions were put in place during the first wave of the pandemic in home care organisations in Region Stockholm and what the managers thought could or should have been done to handle the situation.

### Setting

This study targets home care organisations in three different municipalities in Region Stockholm, the capital of Sweden. Stockholm was targeted as it was particularly hard hit by the pandemic. The three different municipalities (Stockholm City, Södertälje and Norrtälje) were purposively selected to provide variation in geographical location, population density and care system.

In Stockholm City and Södertälje the responsibility for health and social care is divided between the region and the municipalities. The Region is responsible for providing healthcare (as well as home-based healthcare) and the municipalities are responsible for providing social care, such as personal care and services in the home, personal assistance and institutional care. The difference between the two municipalities lies in the number of people in the municipality and the subsequent number of providers of home care in each municipality. In Stockholm municipality there are 184 providers of home care services (155 private providers and 29 public providers). In Södertälje there are only 4 (3 private providers and 1 public provider). In Sweden, the same rules and regulations apply to both private and public providers and there are no extra costs for the clients for choosing a private provider.

The main provider of both health and social care in Norrtälje is the public provider TioHundra. However, there are also four private providers of home care services in Norrtälje. A difference in Norrtälje compared to Stockholm and Södertälje is that all home care providers also offer home healthcare services, which might be of importance in a pandemic situation since health- and social care workers are in the same organisation. The integrated care organisation in Norrtälje might also be able to organise and relocate staff more rapidly, integrate and coordinate measures against the spread of the virus in both health and social care, coordinate the personal protective equipment (PPE) more effectively and more efficiently spread important information to all levels in the organisation.

### Interview guide

An interview topic guide was developed inspired by existing literature [17–19]. The interview questions covered access and use of resources during the pandemic and actions taken to prevent and control spread of Covid-19. Each informant was also asked to describe their job role, the work and service that the provider delivers daily, number of clients and number of staff, to provide an understanding of the home care organisation. A pilot interview was undertaken followed by minor changes to the order of the questions.

### Sampling strategy

Twelve public and private home care providers listed on the website of each municipality were sampled. The managers of the home care providers were telephoned by researcher AL and informed about the study and asked to participate. Five declined participation due to time constraints. The remaining seven agreed to participate in one-to-one interviews. There were three participants from Norrtälje (two from private providers and one from the public provider), two participants from Södertälje (both from private providers), and two participants from Stockholm (one public, one private). All participants were informed about their rights to interrupt and withdraw at any time during the interview or after the interview as long as no results have been published. Apart from information about the study and their rights, participants were also given opportunities to ask questions via email and telephone prior to providing consent. All participant consented to participation in the study. Researcher AL conducted the interviews in Swedish by telephone or Zoom between 5th and 30th November 2020 based on the informant’s preference and availability. Each interview lasted about 1 h. The interviews were audio recorded and transcribed verbatim. After each interview, field notes were taken and identifiable information in the transcripts was anonymized.

### Description of participants

Two of the participants were men and five were women. The age range was 30–64 and five of the participants were below 40 years. Three of the participants were educated as health care professionals, one as social works and three had more administrative backgrounds. Two of the participants had started their current position in march 2020, but both had previous experience in similar positions within the company they worked in. Four of the participants had more than 5 years of experience as managers within home care. Two had more than 10 years of experience. Most of the participants have hands-on experience with providing home care to older adults either previously in their carrier or as par.

### Data analyses

The data analysis of the transcribed interviews was conducted by researchers JA and AL using content analysis as this data analysis method is suitable for descriptive studies [14] and allows for the research question to be depicted after the data have been collected [20]. The two researchers read the interview transcripts independently to achieve immersion and to identify codes. Main thoughts were written down, compared and organized into potential categories. The categories were repeatedly discussed and revised before entered into the coding software NVivo. Two transcribed interviews were then coded by the two researchers together and the rest of the interviews were coded by JA. For the translation of categories and quotes into English, choice of words and synonyms to make the translations as accurate as possible, were discussed. The entire research team contributed to the interpretations of the findings.

### Trustworthiness

JA and AL have several years of experience in ageing research including qualitative methods. AL is a skilled interviewer and has extensive experience of conducting semi-structured interviews with healthcare professionals. Field notes and analytical memos were taken before and during the interview phase. Investigator triangulation have been used through out the analyses process and the results have been discussed with researchers within the field of social gerontology with experience of both qualitative and quantitative research methods. The manuscript have been prepared using the Standards for Reporting Qualitative Research [21].

## Results

The participants’ answers on how the spread of the Covid-19 infection was managed in home care services, were divided into three main themes: Actions taken to handle the spread of the virus, Insecurity and anxiety, and Prerequisites and suggestions on how Covid-19 was and could have been tackled (see Table 1). Codes related to the theme “Actions taken to handle the spread of the virus” focused on the initatives and actions taken to try to minimise the spread of the virus, both among the care workes and among the clients. The second theme, “Insecurity and anxiety”, covered codes related to both care workers and clients anxiety about working and getting care in a pandemic situation, as well as the insecurity that conflicting and constantly changing guidelines inflicted on the managers, care workers and clients. The third theme “Prerequisites and suggestions on how Covid-19 was and could have been tackled” covered suggestions both on improvements within the care organisation and improvements related to a higher level organisation and regulation of the care system.

**Table 1:** Descriptions of themes and codes

Themes	Codes	Descriptions
Actions taken to handle the spread of the virus	Organisational changes	Changes made in how the organisation worked and in how the service tasked was managed
	Access to personal protective equipment (PPE) and guidelines for use of PPE	Participants’ experiences of access to PPE and reasoning about the use of PPE in relation to the official guidelines
	Other internal guidelines	Internal guidelines and regulations that were introduced to avoid spread of infection e.g. temperature checks and ban of visitors
	New recruitments	New recruitments of staff due to shortage of personnel
	Internal education	Internal educational sessions in e.g. basic hygiene routines,
Insecurity and anxiety	Employee anxiety and support	Anxiety experienced by participants or their co-workers, and support to staff
	Experience of external information and guidelines	Handling the flow of information and sometimes conflicting guidelines from authorities
	Anxiety among clients	The participants’ perceptions of clients’ anxiety towards getting infected through care services
Prerequisites and suggestions for how Covid-19 was and could have been tackled	Inside the organisation	Thoughts on how the organisation could have better tackled Covid-19
	Outside the organisation	Thoughts on how outside factors influenced the organisations’ possibility to tackle Covid-19

### Actions to handle the spread of the virus

#### Organisational changes

All participants reported on several organisational changes of home care services that were introduced to minimise the risk of spreading the virus both among clients and employees. First and foremost, different types of cohort care were introduced in all three municipalities studied. In Stockholm and Södertälje, the municipality organised a special Covid-team that visited all clients with diagnosed Covid-19. These teams took over the responsibility of providing care services when a client was diagnosed with Covid-19, irrespectively of the patient’s care provider being private or public.

*“They were very quick to form the covid19 team, “2020” it was called. They were the first among all the municipalities we operate in. And they took care of all those who had covid-19 but actually had another home care provider. They chose to do so, to reduce the spread of the infection. Instead of we going to both healthy customers, and covid19 customers, they took over all those customers until they were healthy, and handed over, back to us, and to other contractors.”*

*Interview 1*

The participants further provided multiple examples of organisational changes that were not externally decided but initiated within the organisations, and therefore differed between organisations. For instance, in Norrtälje, where the home care organisations are also responsible for home healthcare, one of the participants reported that it was decided that the nurses, when possible, should try to avoid going into the older persons’ homes. Instead, medical assessments were conducted remotely by the help of the home care worker [assistant nurse] during their home visit. This was reported to result in fewer home visits and minimised the number of people entering the older persons’ homes. Similarly, some providers replaced visits that did not involve any planned practical work with a telephone call or camera in the older person’s home.

*“[we] have been able to keep down the home visits, you get compensation and so on for services that we do, via telephone and, as a nurse so you can do quite a lot around a patient without being there on site at their home. If you have an assistant nurse who goes to the patient’s home, then you can work with the assistant nurse’s eyes. They can explain, tell you, you ask the assistant nurse questions and then you make your assessment on distance, it’s not as good as when you meet a person physically, but it’s still good”*

*Interview 2*

Some participants reported that administrative personnel were asked to work from home. Some also reported that care workers had the start and finish times of their work shifts slightly delayed to avoid overcrowding in the changing rooms. Additionally, re-prioritising services were something that was mentioned by one of the participants to avoid bringing in new personnel and to facilitate care continuity.

#### Access to and guidelines for use of PPE

The experience of having sufficient access to PPE differed between the participants and differed over time. Almost all participants reported a lack of face masks and visors in the beginning of the pandemic making it impossible to protect staff in every situation.

*“In order to get access to these crisis stocks, there had to be a confirmed or suspected infection. We meant that everyone can be suspected of being infected because there is asymptomatic spread of infection. But that was not the way to reason, because then there would not be enough. So in practice, they [the municipality] meant that our employees would go out and work unprotected, which is a risk both for themselves, their families and for all patients.”*

*Interview 3*

All participants felt they were able to follow the national recommendations at any given time. In the beginning, PPE was only recommended to be used when caring for someone who had tested positive for Covid-19. By the time governmental recommendations changed to use PPE in every situation, the PPE storage had been topped up and were available and used.

*“there was a while there when it was critical but from the time we said we were going to go out with full protective gear, with visors and mouth guards and aprons and this whole thing, there has never been a day when there hasn’t been enough…….once we got the directives that it is full protective clothing that applies then we have had full protective clothing. When we had a shortage, when we failed in how the staff protected themselves, it was before we knew how to go about and where the seriousness lay, when the directives rather were that you should keep your distance and only wear a visor in case of symptoms, that’s what you can see in retrospect that maybe then we had a spread where it went between customer and staff.”*

*Interview 4*

Prior to the introduction of national recommendations/guidelines on the use of face masks and visors, use of PPE varied substantially between the participating organisations. One participant reported that their organisation strictly followed the recommendations by the national authorities which meant that staff used visors but not face masks until such recommendation was implemented nationally. Other organisations introduced face masks earlier to ease anxiety among employees and clients.

*“Very early we also got, I think it was in May but I won’t swear to it, so we introduced mouth protection in all nursing. So that the employees were simply given mouth protection so that it was enough to have a mouth protection on each visit. So that they would not be able to spread infection”*

*Interview 2*

A distinct difference between participants from private providers compared to public providers was the internal storage of PPE that many private providers had in addition to the public storage, which made them less dependent on the centralised storage of PPE

*“everyone has faced the same lack of protective gear, it’s just that we had the opportunity to supplement by portioning out from our own stock as well”*

*Interview 5*

In contrast, participants from public providers reported having to rely on the storage allocated to the municipality. Especially in Stockholm municipality the participants reported about difficulties with the common storage of PPE and hygiene products.

*‘In the beginning it was in short supply, absolutely. There was a backlog from the supplier, and there were no visors to collect, no mouth guards either, that’s how it was all over the world. I know that the administration sat and assembled some form of visor. But then the City started up some kind of emergency stock, and it worked great, but it was also different. I always received when I ordered, while another unit never received. I don’t know how they did it. So I ordered loads here, and then we distributed it….*

*Interviewer: did you portion it out on the units?*

*If I ordered 100 visors, maybe I got 50, but it was still 50. And maybe I got more surface disinfection, and then there was a nursing home, because there I have a contact, they got exactly the opposite. Maybe they got 200 visors, but no surface disinfection, so we switched with each other.*

*Interview 6*

In Norrtälje, it was decided to combine PPE allocated to the region and the municipality into one centralised storage. Both regional and municipal healthcare and social care providers had access to this storage. This enabled the municipal public home care provider to access PPE from the start. Additionally, participants from Norrtälje emphasized the usefulness of working closely with nurses as part of the integrated home health and social care system, facilitating the training in how to use PPE. Frequent contact with the medical doctor in chief further facilitated efficient use of the PPE available.

*“With our somewhat unique position as a healthcare company in both the region and the municipality, we quite quickly got access to the region’s protective equipment stock. We have never really been without protective equipment. We got hold of the visor quite quickly, we got hold of mouth guards quite quickly. Then what to use and when has varied a bit, but then we have been able to discuss it with with, for example, chief physicians.”*

*Interview 7*

#### Other internal guidelines

Several internal guidelines and regulations beyond the PPE regulations were introduced both in home care services and in the care homes, however the guidelines differed between organisations. For instance, most participants reported that they had introduced digital internal meetings both on manager level and for meetings with care workers. Another example referred to internal guidelines restricting the maximum number of people [5] in the same room at the same time. Several participants further reported that they had introduced a ban for visitors to enter the organisation’s premisses before such national guideline for external visitors was implemented. Other internal guidelines reported included introducing body temperature checks of all employees prior to their work shifts, testing their employees for Covid-19 more widely when this became possible, and initiating infection tracing when one or more employees or clients had been diagnosed with Covid-19.

#### New recruitments

Whilst some participants reported that their organisations had employed new personnel on permanent job contracts, most participants reported hiring staff on hourly paid contracts. Many of the new co-workers had experience of working in hotels, restaurants or shops of which some also had experience of working in the care sector.

*“In home care there are different skills that are good to have, of course healthcare skills are good, but above all, more than anything else, it is being service-oriented and friendly and having your heart in the right place, which is the most important. And now that service personnel in other various areas have been laid off, our recruitment situation has been better.”*

*Interview 2*

In general, the participants thought that new members of staff did a good job, but one respondent had mixed experiences of short-term temporary employees including new recruits who quickly had expressed little interest in care work.

Generally, the participants thought that the new employees paid the same attention to the hygiene and infection control as more experienced staff.

#### Internal education and support

Improved training in hygiene routines, often in liaison with the medically responsible nurse [MAS], was the most reported educational initiative. However, the arrangement of the educational activities differed between organisations. Whilst some organisations provided on-site training in smaller groups, some organisations provided the employees with individual guidance by a nurse, and some organisations provided the training online including pre-recorded videos. Some participants did not report on educational sessions but mentioned that information about hygiene routines had been distributed to employees through various channels including internal mobile app or email.

### Anxiety and insecurity

#### Employee anxiety and support

Most participants mentioned anxiety among staff as something that affected the work situation. They reported that this had been an issue especially in the beginning of the pandemic when little was known about the disease, the guidelines were vague and there was not enough PPE to protect the workers in every situation, unless the workers were going to care for confirmed Covid-19 cases. As access to PPE and the possibility to protect themselves increased, the anxiety levels of their clients decreased.

One respondent reported that they had listened to their employees and following an internal investigation of the different recommendations in Swedish authorities and international organisations like WHO, they had introduced stricter guidelines.

In contrast to other municipalities of Region Stockholm, the care services in Norrtälje are integrated which means that nurses are close colleagues with other care workers. According to participants in Norrtälje, nurses supported care workers who worried about providing home care during the Covid-19 pandemic by sharing their knowledge on how the disease spreads and how to protect themselves and their clients by using PPE appropriately.

*“It is clear that there has been fear, it has been like that in the whole society of course. But I still think that this concept that you have in Norrtälje and in some other municipalities where you have nurses linked to the home care organisations, then you have the competencies to talk about spread of infections and talk about how to protect yourself against it and how to protect the customers and so on.”*

*Interview 2*

#### Experience of external information and guidelines

Many participants experienced the information provided by various authorities in the beginning of the pandemic to be vast and to some extent contradictory. The latter was particularly an issue they considered to be related to conflicting messages regarding national guidelines on use of face masks, making it difficult for the participants to know how to act. Also, different municipalities introduced different regulations at different times, resulting in employees being worried about whether the regulations in their organisation were sufficient.

*Some guidelines overlapped, some said different things. I think the municipality has been clear in what information that concerns, or that comes from them, because it has been a big problem in the organisation that you hear something on TV with Anders Tegnell [the state epidemiologist] who has said something at a press conference, Aftonbladet [Swedish newspaper] writes something. Stockholm city says one thing, the region says another. We have had to do a lot of work sifting through which information we believe the employees must relate to.*

*Interview 5*

In Norrtälje, participants reported that the municipality collated information based on relevance before it was distributed. In contrast, some other participants reported passing on all information received, in order not to miss passing on important information. They also reported that the information overload had been difficult for themselves and employees to digest.

*It was hard, I remember, because there was so much uncertainty, and maybe not all the information was directed at me, but it felt more like: oh shit, now I got some directive here, if I email these out, then I’ve done my part, you know? Because then I have my back free. But it becomes very difficult for those at the bottom of the chain.*

*Interview 6*

Some participants in Stockholm and Södertälje had experienced difficulties in reaching out to foreign-born employees who speak very little Swedish due to lack of information from the Swedish authorities in other languages. One of the participants mentioned that foreign-born employees, who also followed the situation in their home countries, worried the most. According to this participant, foreign-born employees felt insecure and questioned the Swedish guidelines as they did not match the guidelines in their home countries.

*Above all, I see that, where Swedish is not the mother tongue, there has been a greater concern. It has been more difficult for us as employers to convey information given that everything [information] is from the regions and the Public Health Agency and so it has not been available in different languages.*

*Interview 5*

#### Anxiety among clients

Most of the participants reported anxiety among the older people cared for, particularly in the beginning of the pandemic before guidelines and PPE had been fully introduced. It was reported that due to anxiety, some clients suspended their home care service.

*Yes. In the beginning, I can say it was very scary. Nobody knew anything. There was no material. Fear… absolutely… high and low, both with our customers, their relatives and of course our employees. In that it was so uncertain how it infects them, but there was a lack of clarity, I can probably sum it up with that.*

*Interview 6*

### Prerequisites and suggestions on how Covid-19 was and could have been tackled

#### Inside the organisation

In general, the participants felt that they had done what they could in relation to the prerequisites they were given. However, some participants expressed that there is room for internal organisational improvement. This included one participant who felt that they had gone back to a pre-pandemic situation too soon after the summer of 2020, considering that an increased spread started again in the fall. Additionally, the dissemination of information to employees within the organisation was mentioned as an area that could have been improved, including that senior staff should have sifted through the information instead of forwarding everything ‘unedited’. It was also thought that the information could have been disseminated in a way that would have been easier for the employees to understand.

Several of the participants mentioned that more comprehensive testing of the care workers could have reduced the risk of infection for clients.

*“..there you probably have something that could have been much, much better. It is the testing of care staff. I would like it to be, not just because you have symptoms, but staff in elderly care should be tested widely with regularity to catch the asymptomatic cases.”*

*Interview 2*

#### Outside the organisation

Some of the participants also shared their thoughts on factors outside the organisation that could possibly have reduced the anxiety among care workers and facilitated quicker adaption to the crisis. Two of the participants requested better crisis management, both in relation to a plan for how to work in a pandemic situation and in relation to developing an emergency stock for hygiene products and PPE.

*... there must be preparedness to adapt quickly if something similar happened again, that didn’t exist before. Finding ways to… a contingency plan perhaps.*

*Interview 6*

Several participants requested clearer recommendations on use of PPE from an earlier stage. They felt that there was too much information and too many changes in very short time. Also, infection detection of clients and better collaboration with primary care was believed to decrease the spread among clients.

One of the participants thought that Stockholm municipality should take more responsibility for the regulations and decisions made in relation to how the work was performed during the pandemic (e.g. what type of PPE that was used in what situations), instead of leaving the responsibility to the care workers to explain clients and relatives.

Another participant reported that the reimbursement system favours multiple visits, making it a barrier for organising the care in a safer manner.

*“…contracts in home healthcare, which steer towards completely wrong things. This means that we must make as many visits as possible, which is totally life-threatening in this pandemic. And I don’t think that has been good.”*

*Interview 3*

## Discussion

The aim of this study was to understand how the spread of the Covid-19 infection was managed in home care services in Region Stockholm during the early phase of the pandemic, what actions were taken to minimise the spread of the disease among clients and staff, and what could have been done differently seen in retrospective. Three themes were identified from the interviews covering “Actions taken to handle the spread of the virus”, “Insecurity and anxiety”, and “Prerequisites and suggestions for how Covid-19 was and could have been tackled”.

A main finding of this study is that there was no single common strategy on how to handle the new virus followed by all municipalities and organisations. Instead, the home care organisations in each municipality developed their own strategies during the first wave. This finding corresponds to a previous evaluation of the Swedish Covid-19 strategy by the Corona Commission, a commission appointed by the Swedish government to review the Swedish Covid-19 strategy [2, 4, 5]. Not only did the commission conclude that the Swedish health and social care was ill-prepared and poorly equipped to cope with the increased pressure caused by the pandemic, it also highlights that particularly staff employed in social care were largely left to themselves to tackle the crisis [2]. This could be put in relation to the multi-professional expertise considered needed to make interdependent decisions typical for the pandemic [22].

Although there was a lack of official national and regional regulations, many of the initiatives introduced in the municipalities and organisations turned out to be similar. Similarities across municipalities and organisations included the introduction of specific teams dedicated to care for clients with diagnosed Covid-19, by some participants referred to as cohort care, “cohorting”. Generally, cohorting was used in long-term care homes during the pandemic and evidence suggests that cohorting might decrease the risk of being infected although evidence is very uncertain, according to a recent review [23]. To the best of our knowledge there are no studies on cohorting in organisations providing home care including allocating designated care workers to visit only Covid-19 patients to limit the spread of the virus to other clients. This might be of particular interest to the Swedish context as lockdown was not implemented. Another similarity among the participants in this study was the experience of having difficulties getting sufficient PPE during the first month of the pandemic. The nationwide lack of PPE has also been criticised by the Corona Commission [2]. Yet, PPE shortage among home care providers was not unique for Greater Stockholm and has also been reported in studies from, for instance, Norway [8] and the USA [9, 10, 24, 25]. Nevertheless, this study also demonstrates differences in PPE access between public and private home care providers as participants reported that private providers tended to have access to both the public and their own internal storage of PPE, which was considered an advantage in the initial phase of the pandemic. Whilst none of the participants reported having struggled to meet the guidelines for PPE use, they sometimes struggled to get hold of the amount of type of PPE that they would have preferred to procure, a finding that supports previous American studies [10]. Most participants reported that their organisation had introduced additional guidelines and subsequently either used more PPE than just the basics such as face mask and gloves or used PPE in more situations than recommended in the national official guidelines. The use of face masks was debated in Sweden. For instance, the Swedish Public Health Agency did not recommend use of face mask in home care in the initial phase of the pandemic due to lack of evidence of infection control [26, 27]. Thus, in the beginning of the pandemic PPE was only to be used when visiting clients with a confirmed Covid-19 diagnose – in all other situations basic hygiene routines should be applied in combination with as much social distancing as possible. The recommendations on use of PPE in social care gradually changed to also include use of PPE even when visiting clients with symptoms, and eventually to be used in all situations that involved providing personal care.

The second theme about anxiety and insecurity was not initially part of the interview guide, however, most of the participants lifted the ever changing and sometimes conflicting regulations and guidelines as inflicting unnecessary anxiety and insecurity among both staff and clients. Several participants asked for clearer and more conforming communication from the authorities. This sub-finding supports a Norwegian study in which managers of home care services reported constantly changing guidelines and routines to be a main challenge causing the managers to make trade-offs between care demand and guidelines [8]. Whilst experienced managers are used to make various trade-offs, this study contributes with additional aspects that made the situations particularly challenging such as substantial numbers of staff with language barriers. To better manage future pandemics, the participants suggested that guidelines and updates from authorities disseminated to them as managers and then passed on to staff should be trimmed to give priority to the most important information.

Statistics have shown that Norrtälje municipality did have a lower mortality rate among their older population than most other municipalities in Region Stockholm [28, 29]. Though there might be many explanations for this, the integrated care system of Norrtälje has been considered a possible explanation [30]. Indeed, this study has found that the integrated care system in Norrtälje has some advantages that might be of importance in a situation like the Covid-19 pandemic. This included joint organisation of health and social care providing the possibility to communicate with healthcare professionals on management level, discussing best practice, and sharing PPE and hygiene products between health and social care across the municipality to ensure that PPE was used where it was needed the most. The fact that home care organisations in Norrtälje provide both home healthcare and home care services also enabled nurses in the organisations to help disseminate and interpret relevant information as well as educate care workers in hygiene procedures.

### Strengths and limitations

Strengths of the study include that the interviews were conducted with managers of both public and private home care providers from three different municipalities in Region Stockholm. All interviews were further conducted within 4 weeks, which is of importance in terms of participants referring to the same time period capturing the early phase of the pandemic including the first wave. Limitations include the small number of interviews making it difficult to demonstrate data saturation and consistency between the data presented and the summary of the findings. It also makes it difficult to generalise the findings. However, the findings support existing literature, indicating qualitative validity of the study. Also, the concept of information power in qualitative research suggests that data from a smaller number of interviews might be considered adequate if the research topic is little explored [31]. Finally, the participants were all managers which is likely to have provided a one-sided story as perceptions on care are likely to differ depending on the occupational hierarchy.

### Implications for practice

This study suggests some implications for practice. First, the findings from this study stress the importance of a general main strategy for handling future pandemics so that each municipality does not have to develop and decide upon their own local strategies. In practice, this requires home care to be better incorporated into future public health pandemic plans.

Second, clear and concordant communication from authorities and a more pronounced division of responsibility from the municipalities would have helped the home care providers in the initial phase of the pandemic. In the future, information on recommendations and guidelines needs to be more structured, concise and translated into relevant languages to make it easier to read, follow and digest. This could also have a positive impact on unnecessary anxiety among staff and clients.

Lastly, this study suggests that having a closer communication between health and social care providers may positively influence the coordination and management during a pandemic. More research is needed to understand the effect of integrated care in managing the challenges of providing care for older people during a pandemic.

## Conclusions

This study showed that there was no single strategy on how to handle Covid-19 among the participating home care organisations, although there were similarities. Designated Covid-19 teams were introduced in almost all organisations and all participants mentioned having introduced stricter regulations regarding use of personal protective equipment (PPE) than the official guidelines.

Otherwise, the strategies varied between organisations e.g., related to education on hygiene routines, recruitments of staff, restricting number of individuals in the same room, ban on visitors in the administrative facilities, testing of employees and limiting the number of visits to clients. PPE shortage was considered a major source of uncertainty and fear, and points to an area of improvements for handling future pandemics. Participants in Norrtälje thought it was useful that the municipality and region combined and distributed PPE according to needs from the very beginning. Greater stocks of both PPE and disinfectants were mentioned by the participants as a future recommendation.

Social care workers providing home care to older persons are an important group in preventing the spread of infectious diseases like Covid-19. For better readiness and preparedness for future pandemics, municipal home care services would need stocks of PPE, clear guidelines and more training on how to reduce dissemination of disease. Ways to achieve closer communication between health and social care providers should also be investigated.

### Supplementary Information

**Additional file 1.** Interview Guide - Home Care.

## Data Availability

The interview data analysed in this study are not publicly available, as the respondents have been promised anonymity and sharing the interview data would compromise anonymity. De-identified data is available from the corresponding author upon reasonable request.
