# Supplementary material for: How did providers of home care for older adults manage the early phase of the Covid-19 pandemic? A qualitative case study of managers’ experiences in Region Stockholm
Source: BMC Health Serv Res. 2023 Oct 27;23:1173. doi: 10.1186/s12913-023-10173-8 (PMC10612274; doi:10.1186/s12913-023-10173-8)
Supplement: Supplementary file 1 — Additional file 1. Interview Guide - Home Care. [file 12913_2023_10173_MOESM1_ESM.docx]

Interview Guide - Home Care

**General Questions about the Organization**

1. Briefly describe how you as a home care organisation work with the municipality.
2. How do you experience the collaboration with the municipality and the healthcare services?
   1. How do you collaborate with the municipality and healthcare services?
   2. At what levels/levels of management does this collaboration occur?
   3. How does it work practically in terms of contacts and meetings? How often and with whom?
   4. How do you feel this collaboration is functioning?
   5. Who should collaborate with each other? How? Why?
3. What authority do managers/unit leaders have regarding decision-making on protective measures, staff routines, etc.? Can you provide examples?
4. How many elderly individuals do you care for?
5. How many employees do you have?
   1. How many are permanent employees (full-time/part-time) and how many are temporary staff?
   2. What percentage have qualifications as nursing assistants or higher educational levels?
   3. How many elderly individuals does each employee care for?
6. Do permanent employees seem to have a better understanding of how disease transmission occurs compared to temporary staff/other industries?
7. Where did the infection come from? Were any employees infected? How did they experience the situation?
8. Have you/are you working with a medicinskt ansvarig sjuksköterska (medical responsible nurse)?

**Discovery of COVID-19 in the Municipality**

1. When COVID-19 was first detected in an elderly individual in the municipality, what measures, if any, did you take?
   1. What was done to protect the elderly in their homes?
   2. What was done to protect the staff?
2. To what extent did you try to implement guidelines from the Public Health Agency? Can you provide examples?

**Resources During the Pandemic**

1. How was the availability of materials and personal protective equipment during the spring of 2020?
   1. What factors influenced availability?
2. Did the staff use personal protective equipment (face shields, masks, plastic aprons)?
   1. If yes, to what extent (e.g., was all staff fully protected at all times), and from when?

**Measures and Collaboration During the Pandemic**

1. In hindsight, which of the measures taken were effective in reducing the spread of the virus? When were they implemented?
2. In hindsight, what other measures should have been taken (but weren't)? When?
   1. What do you believe influenced the decision to implement or not implement these measures?
3. How do you think the organization has generally responded to COVID-19?
4. What do you consider the organization's weakest point during the spring 2020 pandemic?
5. Has COVID-19 led to changes in the organization's structure or operations?
   1. If yes, how?
6. How have you collaborated with the municipality during the pandemic?
   1. What has worked well?
   2. What do you believe contributed to its success?
   3. What has not worked as well?
   4. What do you believe contributed to its shortcomings?

Do you have any additional thoughts on your organization's or the municipality's response during the coronavirus pandemic?

a. If yes, could the issues you raise have been influenced by your organization's/municipality's structure?

Anything else to add?
